# Annexin V and anti-Annexin V antibodies: two interesting aspects in acute myocardial infarction

**DOI:** 10.1186/1477-9560-7-13

**Published:** 2009-07-21

**Authors:** Mohammad Shojaie, Abdoreza Sotoodah, Shohre Roozmeh, Ensieh Kholoosi, Samira Dana

**Affiliations:** 1 Department of Cardiology, Jahrom University of Medical science, Jahrom, Iran; 2 Department of Immunology, Jahrom University of Medical science, Jahrom, Iran; 3 Department of Medicine, Jahrom University of Medical science, Jahrom, Iran

## Abstract

**Background:**

Myocardial infarction is the combined result of environmental factors and personal predispositions. Prothrombotic factors might play an important role in this phenomenon. Annexin V (ANV) is a calcium-dependent glycoprotein widely present in various tissues exerting a potent anticoagulant effect in vitro by reducing plaque adhesion and aggregation. Anti-annexin V antibodies (aANVAs) are detected in various diseases like rheumatoid arthritis, systemic lupus erythematosus and anti-phospholipid antibody syndrome. The study of ANV in Acute Myocardial Infarction (AMI) might shed light on hypercoagulability mechanisms in the pathogenesis of acute coronary syndromes. This study was conducted to investigate the association of plasma ANV, aANVAs and anti-cardiolipin antibodies (aCLAs) with AMI.

**Methods:**

This study recruited 45 patients with the diagnosis of AMI according to WHO criteria in their first 24 hours of admission. 36 matched individuals were studied as the control group with normal coronary artery angiography. Plasma levels of ANV, aANVAs and aCLAs were determined by enzyme-linked immunosorbent assay and the results were compared.

**Results:**

Plasma ANV levels in the patients with AMI on admission were significantly lower than those in the control group (p = 0.002). Positive test for aANVAs were found to be present in a significant number of our patients (p = 0.004). The studied groups were similar in their rate of patients with positive aCLAs tests. ANV, aANVAs and aCLAs were not correlated with hypertension, diabetes mellitus, hyperlipidemia, sex, age and smoking.

**Conclusion:**

Our findings suggest that low plasma ANV levels along with positive aANVAs tests in patients with AMI are indicative of hypercoagulable state that is not related to the traditional cardiovascular risk factors.

## Introduction

Myocardial infarction (MI) is the combined result of environmental factors and personal predispositions [1]. Prothrombotic factors may play a more important role in these patients. Various prothrombotic factors and markers of endothelial damage have been associated with an increased risk of myocardial infarction e.g. fibrinogen [2], tissue plasminogen activator (t-PA) [2-4] and the von-Willebrand factor [2,3].

Annexin V (ANV) is a calcium-dependent glycoprotein with a potent anticoagulant capacity in vitro [5] (mainly as a result of its negatively charged membrane phospholipids), and capable of inhibiting the prothrombinase and Tensa complexes to reduce plaque adhesion and aggregation [6]. Circulating ANV can be released from the cells of the vascular wall (endothelial cells, smooth muscle cells) or from secretor cells of the spleen and liver. Once it is in the plasma, it binds to blood cells (platelets and erythrocytes) or to endothelial cells [7].

In addition, ANV possesses a high apoptotic cell affinity as these cells produce a large amount of phospholipids, particularly phosphatydilserine [6]. ANV appears to form an «antithrombotic shield» around the phospholipids, displacing their coagulation factors [8]. Furthermore, ANV has been shown to be an effective inhibitor of thrombus formation in a venous and arterial thrombus model and in vitro perfusion models. [9,10]. ANV binds to the phosphatidylserine inhibiting the pro-coagulant and pro-inflammatory activities of the dying cell. It is considered as an unspecific apoptosis marker [11].

The complex of phosphatidylserine and extracellular ANV provides an antigenic stimulation for antibody production. Anti-annexin V antibodies (aANVAs) were detected in various abnormalities like rheumatoid arthritis (RA), systemic lupus erythematosus (SLE) [12-15], anti-phospholipid antibody (APA) syndrome [16] and in cerebrovascular disease [17].

This antibody has been associated with the occurrence of thrombotic events and/or recurrent abortions in patients with SLE and APA syndrome as well as digital ischemia in patients with systemic sclerosis (SSc). Moreover, it is suspected that aANVAs may interfere with annexin V function(s) and exert a detrimental role leading to thrombosis and/or vascular occlusion [18]. It has been proposed that APA syndrome may cause thrombotic events by means of inhibition of ANV binding and resistance to ANV anticoagulant activity [19].

ANV is widely used as a tool in apoptosis research [20], but its physiological role has not been studied extensively in relation to vascular biology. Few controversial studies of aANVAs and acute coronary syndromes exist [20-22]. The investigation of ANV, aANVAs and anti-cardiolipin antibodies (aCLAs) in MI might shed light on hypercoagulability mechanisms in the pathogenesis of acute coronary syndromes.

Our goal was to study the plasma level of ANV, aANVAs and aCLAs in patients who developed acute myocardial infarction, and to analyze their relationship with traditional cardiovascular risk factors.

## Methods

### Subjects

This case-control study recruited 45 consecutive patients with acute myocardial infarction (AMI) including 31 men and 14 women with the mean age of 62.7 ± 13.1 years old who were taken to the emergency room of Peymanieh Hospital (Jahrom, Iran) with the chief complaint of chest pain from Feb 2007 to May 2008.

We also selected 36 individuals that referred to the emergency room with chest pain with normal coronary angiography as our control group and matched them for age, sex and other CAD risk factors such as hypertension (HTN), diabetes mellitus (DM) and hyperlipidemia (HLP).

The study protocol was approved by research ethics committee of Jahrom University of Medical Sciences and informed consents were obtained from all participants before enrollment.

A questionnaire including information about the past medical and drug history (HTN, HLP, DM, smoking, chronic diseases such as collagen vascular diseases and asthma), family history of coronary artery disease (CAD) and demographic information was completed for each patient.

The exclusion criteria were the presence of severe liver disease, malignancy, recent cardiac surgery, angioplasty, stable or unstable angina, receiving of anticoagulant drugs, hemolysis, pregnancy loss, history of deep vein or arterial thrombosis, inflammatory and rheumatologic diseases such as collagen vascular disease, SLE and APA syndrome.

### Definitions

AMI was defined as chest pain lasting more than 30 minutes accompanied by ischemic electrocardiographic changes and was confirmed by the presence of total creatinine phosphokinase (CPK) or MB fraction levels of more than twice the upper normal limit [23,24]. The absence of any narrowing in coronary artery diameter was considered as normal coronary angiography.

Blood pressure was measured two times in sitting position after 5 minutes of rest using a mercury sphygmomanometer. Hypertension was defined as blood pressure more than 130/85 mmHg or use of any antihypertensive medication [25]. DM was defined by a physician's diagnosis, a fasting plasma glucose level of ≥126 mg/dl or use of diabetic medications [26]. Echocardiography was done for all patients during their hospital stay by one cardiologist. Ejection fraction (EF) is defined to be normal (>55%), mild (45–54%), moderate (30–44%) and (>30%) severe LV systolic dysfunction [27].

### Laboratory analysis

Fasting levels of plasma total cholesterol, High density lipoprotein (HDL) cholesterol, low density lipoprotein cholesterol (LDL), and triglycerides (TG) were measured in Research Laboratory of Jahrom Medical University. Total cholesterol and triglyceride levels were measured by enzymatic techniques using a Selectra E biochromatic analyzer. HDL and LDL cholesterol level was measured after glucose levels were measured by the glucose oxidase method. CPK were measured by an enzymatic method.

Blood samples (5 cc) were obtained by venipuncture from the patients immediately after admission before starting any IV medications by trained staff and for lipid profile and fasting blood sugar at the first 24 hours of AMI after 12 hours of fasting. In control subjects all blood sample were obtained after 12 hours of fasting then plasma was separated and frozen at -70°C for later processing. Level of circulating annexin V and anti-annexin V and anti-cardiolipin IgG antibodies were determined by enzyme linked immunosorbent assay (ELISA) using high-sensitivity commercial kits (Aeskulisa, REF: 7204, Germany for aCLAs, Bender Medsystems company, Cat. No.: BMS252, Austria for annexin V and Medsystems Company, Cat. No.: BMS247, Austria for aANVAs) according to manufacturer's recommendations.

The detection range for annexin V was indicated as0.2 to20 ng/mL. We consider aCLAs level above 15 ng/mL and aANVAs level above1.18 ng/mL as positive results.

### Statistical analysis

Statistical analysis was performed by SPSS (version 15; SPSS, Inc., Chicago, IL). Data were expressed as mean ± 1 SD. Continuous variables with little-to-mild skewness were summarized as mean ± SD and compared using Student's *t*-test. Discrete variables were presented as frequencies and group percentages. Nominal variables were tested with Pearson's χ^2 ^test and Binary variables were tested with the chi square test. Pearson correlation coefficients were calculated to evaluate unadjusted (univariate) associations between ANV and other variables. Generalized Linear Models were used to adjust smocking between two groups. All tests were two-tailed with a 0.05 type I error rate. ANOVA and Kruskal-Wallis test were used to evaluate association of ANV, aANVAs and aCLAs with different type of AMI and EF.

## Results

**Figure 1:**
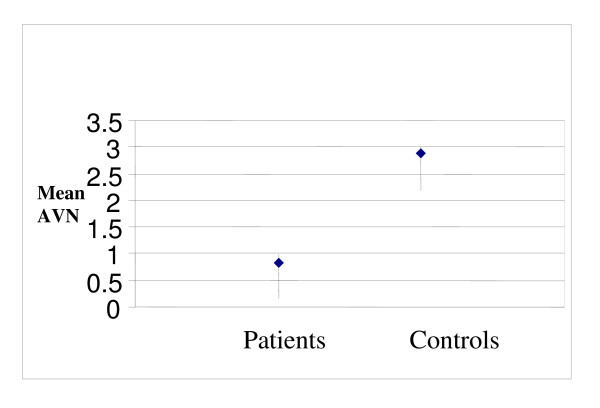
**Plasma ANV in patients who had acute myocardial infarction and in controls 0.83 ± 0.77 ng/ml vs 4.12 ± 2.88 ng/ml; p = 0.002**.

**Figure 2:**
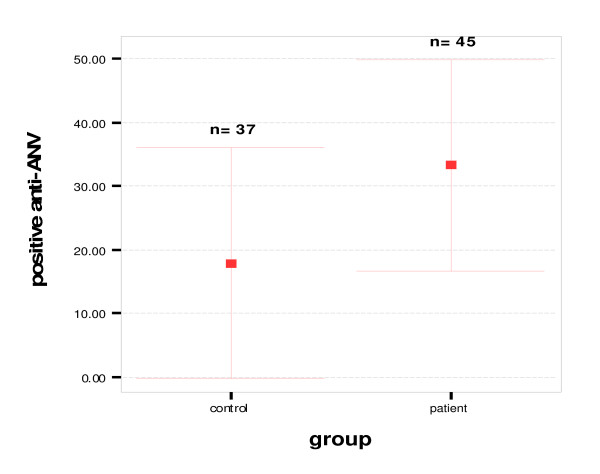
**Positive anti-ANV antibodies in patients who had an acute myocardial infarction and in controls, 20(45.5%) vs. 6(15.8%), p = 0.004**.

The demographic and clinical characteristics of the study groups, as well as laboratory variables are shown in Table 1. In patient group 6 cases (14%) had non-ST elevation MI (NSTEMI) and 37 (86%) had ST elevation MI (STEMI). There was no significant difference between the two groups regarding the following variables: age, sex, presence of HTN, DM, LDL, HDL, total cholesterol and TG. Plasma ANV levels in patients with AMI on admission were significantly lower than those in the control group (0.83 ± 0.77 ng/ml vs. 4.12 ± 2.88 ng/ml, p = 0.002) (Fig 1). Also, our patients had significantly more positive anti-annexin V antibody results than the control group [20(45.5%) vs. 6(15.8%), p = 0.004] (Fig 2) but no difference was found in the aCLAs test results between the two groups (Table 1).

**Table 1:** Demographic and clinical characteristics of the study groups

**Variable**	**Case group** **n = 45**	**Control group** **n = 36**	**P-value**
**Age**	62.7 ± 13	60.1 ± 11.9	0.38
**Male, n (%)**	31 (68.9%)	18 (50%)	0.08
**Current smoker, n(%)**	11 (24.4%)	2 (5.6%)	0.02 *
**HTN, n(%)**	8 (17.8%)	11 (30.6%)	0.18
**Type 1 DM, n (%)**	2 (4.4%)	4(11.1%)	0.4
**Type 2 DM, n (%)**	7(5.6%)	4(11.1%)	0.56
**Total Cholesterol (mg/dL)**	189.2 ± 43.7	176.3 ± 32.3	0.14
**LDL-C (mg/dL)**	112.8 ± 35.6	106 ± 28.7	0.36
**HDL-C (mg/dL)**	46.2 ± 11.4	42.3 ± 10	0.11
**LDL/HDL ratio**	4.23	4.24	0.96
**Triglyceride (mg/dL)**	146.1 ± 97.6	153 ± 105.5	0.76
**ANV (ng/mL)**	0.84 ± 0.93	4.12 ± 2.88	0.003*
**aANVAs(ng/mL)**	33.2 ± 55	17.8 ± 54.4	0.2
**Positive aANVAs**	20(45.5%)	6(15.8%)	0.004*
**aCLAs(ng/mL)**	21.7 ± 55.7	13.9 ± 38.1	0.46
**Positive aCLAs**	8(18.6%)	5(13.2%)	0.53

HTN: hypertension, LDL-C: low density lipoprotein-cholesterol, HDL-C: high density lipoprotein-cholesterol, ANV: annexin V, aANVAs: anti annexin V antibodies, aCLAs: anti cardiolipin antibodies

Values are presented as mean ± SD or %.

We examined the association between plasma ANV, aANVAs and aCLAs and selected cardiovascular risk factors. There was not a significant correlation between ANV and the other two antibodies in patients and controls but we found significant correlation between aANVAs and aCLAs in patients (r = 0.69, p = 0.000) and controls (r = -0.91, p = 0.000). Also, we didn't find a significant association between plasma ANV, aANVAs and aCLAs with HTN, Type 1 DM, Type 2 DM, age, sex, LDL, HDL, TG, total cholesterol and adjusted smocking.

We didn't find a statically significant association of plasma ANV, aANVAs and aCLAs with type of MI, LV systolic function (EF) and mortality in our cases and with sex and not with age in all subjects. Plasma ANV level in patients with STEMI and those with NSTEMI were not significantly different (0.75 ± 0.73 μg/ml vs. 1.35 ± 1.47 μg/ml, p = 0.28).

## Discussion

In the present case-control study, in line with previous studies [21], we found that lower plasma ANV levels were associated with AMI among Iranian patients independent of traditional cardiovascular risk factors.

Like our findings, Roldan et al [21] found lower ANV in patients with old MI in comparison with normal subjects being in contrast to others that showed higher than controls levels [20] or reporting levels to be within normal limits [22]. Roldan's study also found that ANV is not correlated with traditional atherosclerotic risk factors. [21] But Oleu [28] considered ANV as a risk factor of premature MI. All that studied patients were under 50 years old which is not comparable to our study.

Cederholm et al [20] showed a significantly higher level of circulating ANV in SLE cases with history of Cerebrovascular Disease (CVD) compared to SLE controls and normal population. This rise could be a result of ANV displacement and/or raised production. Although little is known about the role of circulating ANV it would be possible that ANV contributes to growth of atherosclerotic plaques at a late stage of disease, in which apoptosis, fissures, and microthrombi, as well as endothelial cell activation are common features of the plaque complex facilitating ANV binding to the exposed surfaces. In a previous elegant study, it was demonstrated that arterial thrombosis could be inhibited by recombinant ANV in a rabbit model of carotid artery injury [29].

Kaneko et al [22] and Matsudo et al [30] in two separate studies showed early elevation in plasma ANV levels in the first 6 hour of AMI and a subsequent decrement. They could not provide a clear explanation of this observation. In contrast, we found lower ANV levels in our patients. This discrepancy might be a result of auto antibodies against ANV, displacement of ANV by aCLAs [20], selective ANV binding to the thrombi [31], race differences and/or hypercoagulable states in our patients.

Matsudo and his colleagues [30] found association of AVN levels with prognosis but in our study, we could not find any association between ANV and type of MI, EF and mortality. It seems that ANV could be better considered as a marker of apoptosis. An anecdotal study by Kaneko and his coworkers [22] concluded that ANV is a diagnostic test for AMI but much evidence including ours is to the contrary of such an assumption.

It has been proposed that ANV could play an essential role in the thrombogenic mechanisms of APAs [32]. IgG fractions in patients with APA reduce the ANV levels in trophoblastic and endothelial cell cultures [8], resulting in an increase in the amount of anionic phospholipids capable of initiating coagulation [5]. It is known that APA is associated with hypercoagulability states [33]. IgG fraction from APA patients lowers ANV on non cellular phospholipid surfaces and accelerates plasma coagulation following thrombin generation [19,34]. In our study no correlation was found between APA and AMI but we found strong correlations between aCLAs and aANVAs.

A remarkable clinical finding in our study was the number of positive aANVAs results. In Roldan's study [21] only two patients with positive aANVAs were found but in our study about 45.5% of patients had positive aANVAs being the first study to identify such a high incidence in AMI. These discrepancies may be due to they detected anti ANV IgG only but we detected all antibody subtypes against annexin V and also race differences. Mechanisms raising aANVAs are not completely elucidated but it is proposed that in the context of increased apoptosis, extracellular/membrane ANV might become an antigenic stimulus for specific antibody production [17]. Such antibodies may have a detrimental role interfering with putative functions of ANV [35]. Since there is a lag or latent phase between the initial exposure to an immunogen and detection of antibodies in the circulation, which several days average about 1 week in human [36] and we got our samples in the first day of AMI, it could be concluded that aANVAs have been present in our patients before MI occurrence and are not secondary to it.

The occurrence of auto antibodies to ANV has been described in several pathological disorders encompassing thrombosis mechanisms. For example, aANVAs concentrations are raised significantly in sera of RA patients compared to normal controls [15]. Sugiura and Muro [37] showed that aANVAs correlates with the occurrence of digital ischemia in SSc patients. Thrombosis is increased in APA patients with aANVAs positive [16]. Kaburaki and colleagues [38] found a correlation between the detection of aANVAs and clinical presentations of arterial and/or venous thrombosis, intra-uterine fetal loss and prolonged activated partial thromboplastin time in SLE patients.

In our study, serum ANV level, aANVAs and aCLAs were measured in a single sample. We couldn't followed the patients prospectively and perhaps a prospective cohort study for ANV and aANVAs levels would become more narrative as the next step to elucidate their portray. Gaspersic N et al [39] showed competition of anti-β2 glycoprotein I with ANV for binding to phospholipid but we didn't analyze the presence of anti-β2 glycoprotein I in patients.

## Conclusion

We showed, for the first time, the high incidence of aANVAs in AMI. Our findings further support the notion that ANV has antithrombotic properties. Our data suggest that the low plasma ANV together with high level of aANVAs in patients with AMI may indicate the existence of a hypercoagulable state that does not appear to be related to the traditional cardiovascular risk factors.

## Competing interests

The authors declare that they have no competing interests.

## Authors' contributions

MS had substantial contributions to conception and design and interpretation of data and writing the manuscript. EK and SD had contribution to sampling. AS carried out the biochemical analysis. SR had contributions to data analysis. All authors read and approved the final manuscript.
